# Perceptions of Autism Spectrum Disorder (ASD) Etiology among Parents of Children with ASD

**DOI:** 10.3390/ijerph18136774

**Published:** 2021-06-24

**Authors:** Wei-Ju Chen, Zihan Zhang, Haocen Wang, Tung-Sung Tseng, Ping Ma, Lei-Shih Chen

**Affiliations:** 1 Department of Psychology, The University of Texas Permian Basin, Odessa, TX 79762, USA; chen_w@utpb.edu; 2 Department of Health and Kinesiology, Texas A&M University, College Station, TX 77843, USA; zihan2017@tamu.edu (Z.Z.); hwang472@tamu.edu (H.W.); 3 Health Sciences Center, Behavioral and Community Health Sciences Program, School of Public Health, Louisiana State University, New Orleans, LA 70112, USA; ttseng@lsuhsc.edu; 4 Department of Health Promotion and Community Health Sciences, School of Public Health, Texas A&M University, College Station, TX 77843, USA; pma@tamu.edu

**Keywords:** autism spectrum disorder, parents, perceptions, etiology

## Abstract

Background: Autism spectrum disorder (ASD) is a neurodevelopmental disorder characterized by social communication deficits and restricted or repetitive behaviors. Parental perceptions of the etiology of their child’s ASD can affect provider–client relationships, bonding between parents and their children, and the prognosis, treatment, and management of children with ASD. Thus, this study sought to examine the perceptions of ASD etiology of parents of children with ASD. Methods: Forty-two parents of children diagnosed with ASD were recruited across Texas. Semi-structured interviews were conducted individually. All interviews were recorded and later transcribed verbatim for content analysis utilizing NVivo 12.0 (QSR International, Doncaster, Australia). Results: The content analysis identified the following themes regarding parental perceptions of ASD etiology: Genetic factors (40.5%), environmental factors (31.0%), problems that occurred during pregnancy or delivery (23.8%), vaccinations (16.7%), other health problems (7.1%), parental age at the time of pregnancy (4.8%), and spiritual or religious factors (2.4%). Conclusions: The parental perceptions of ASD etiology were diverse, but several views, such as vaccinations and spiritual or religious factors, were not based on scientific evidence. Health professionals and researchers can use these findings to develop and provide targeted education to parents who have children with ASD. Our findings also support policymakers in developing campaigns designed to increase parental ASD awareness and knowledge.

## 1. Introduction

Autism spectrum disorder (ASD) is a neurodevelopmental disorder characterized by social communication deficits, restricted or repetitive behaviors, and stereotyped interests [1]. The onset of ASD symptoms or behaviors can occur in one of two ways: Early or regressive [2,3]. In early onset, ASD symptoms (e.g., deficits or delay in social and speech development) occur in the first year of life. Children with regressive onset initially exhibit typical development. However, in the second or third year of life, they begin to exhibit ASD symptoms or behaviors accompanied by the loss of previously established social, communicative, and/or motor skills [2]. Researchers have proposed a third onset pattern, plateau, in which children display typical patterns of development in the first year, followed by a slowdown or lack of further development. Although children retain previously acquired skills in plateau onset, their development plateaus with no progress to advanced skills such as language or joint attention [2,3].

Individuals with ASD may be at risk for deficient cognitive abilities, sensory processing anomalies, impaired language development, and other medical conditions (e.g., seizures, sleep disorders, and psychological disorders) [3,4]. Researchers have continued to study the cause of ASD, as findings have the potential to inform ASD diagnosis, treatment, management, and prognosis, help predict or anticipate possible comorbid medical conditions, and increase adherence to intervention and rehabilitation regimens. Furthermore, parents of children with ASD often support ASD etiological research as it helps with family planning and reduces the guilt and anxiety parents feel that is associated with their speculations as to the cause of their child’s ASD [5,6].

Although the exact cause of ASD remains unclear, research on ASD etiology has suggested two main contributing factors: Genetics and the environment. There are also possible interactions between genes and the environment, suggesting that ASD could be a multifactorial disorder [7,8]. Of the two factors, genetics play a larger and more critical role than environmental factors [9,10], as has been shown in many ASD studies, such as those conducted with twins, relatives, and rare syndromes [11,12]. Researchers have identified over 200 genes associated with ASD [13], examined ASD-related chromosomal abnormalities, copy number variants (CNVs), and single-gene disorders [11,14], and reported high heritability of ASD ranging from 80% to 90% [14,15].

Despite the strong genetic evidence provided by ASD etiology research, there are still misconceptions about both ASD and its causes [16]. One of the most significant concerns is about the perceived link between vaccinations and ASD [16,17]. An early event that initiated this misconception was a documentary released in 1982 titled DPT: Vaccine Roulette [18], suggesting a link between the diphtheria, tetanus, and pertussis (DTP) vaccine and the development of neurodevelopmental disorders such as ASD [17]. Another major event was an article published in 1998 by Andrew Wakefield and his colleagues [19] in the Lancet, in which they proposed that the measles, mumps, and rubella (MMR) vaccine was linked to ASD [17,20]. This paper was later retracted by the Lancet in 2010 due to incorrect information, misrepresentation, and ethical violations [17,20]. Though many researchers have studied these claims and have found no evidence of an association between vaccinations and the development of ASD [17,21], these concerns still remain among parents and caregivers [16,22].

Misconceptions about the causes of ASD can negatively influence families affected by ASD by serving as obstacles to proper ASD education and treatment [16,23]. It is therefore of crucial importance to study parental perceptions (and potential misconceptions) of ASD etiology. Not only do parental perceptions of the cause of ASD affect bonding between parents and their children with ASD, they can also influence provider–client relationships and the prognosis, treatment, and management of ASD of their children. In addition, understanding parental perceptions and misperceptions of ASD etiology can help health professionals, researchers, and policymakers better educate and serve families in need [2,24,25].

Although studies have explored parental perceptions of ASD etiology, several gaps remain in this area of research. First, many previous studies utilized surveys/questionnaires and took a quantitative approach to data analysis [24,25,26,27,28,29,30]. Fewer studies have collected more in-depth, qualitative data about the reasons behind parents’ perceived ASD etiology [31,32,33,34,35,36]. Second, only two qualitative studies in this area have been conducted in the United States [31,32]; most related research has been conducted in other countries (e.g., Israel, Australia, and the United Kingdom) [33,34,35,36]. Third, 74.3–93.7% of the participants in the previous research studies were predominantly White/Caucasian [24,28,29,30,31,32], suggesting that minority parents of children with ASD have been understudied. To address these gaps in the existing research, the specific objectives of the present study were to collect in-depth, comprehensive qualitative data to explore U.S. parents’ perceptions of the cause of their child’s ASD and the reasons underlying their perceptions.

## 2. Materials and Methods

### 2.1. Participants and Recruitment

The present study focused on the perceptions of parents whose children were diagnosed with ASD. Prospective participants were recruited from professional health conferences (i.e., Texas Public Health Association Conference, Society for Public Health Education Conference, American Association on Intellectual and Developmental Disabilities Conference, and the Texas Association for Health, Physical Education, Recreation and Dance Annual Convention) and ASD communities (e.g., Easterseals, Autism Speaks, Autism Clinic, Light and Salt Association, Families of Autistic Children Engaged Together for Support [FACETS], and the Center on Disability and Development at Texas A&M University) across Texas. We utilized a snowball sampling technique to create a sample with diverse socioeconomic backgrounds. Parents with at least one child diagnosed with ASD were eligible to participate in this study. All participants who contacted our research team were eligible. Semi-structured interviews were conducted until data saturation was achieved with 42 participants. As an incentive, participating parents each received $50 cash for their participation. The Institutional Review Board at Texas A&M University approved the study protocol.

Of the 42 participating parents, 32 (76.2%) had one child diagnosed with ASD, nine (21.4%) reported two children with ASD, and one (2.4%) participant reported three children diagnosed with ASD. Thirty-two (76.2%) participants were mothers and 10 (23.8%) were fathers. The average age of the participants was 44.3 years (*SD* = 8.5 years; range = 24–58 years). Half (*n* = 21, 50.0%) of the participants self-identified as non-Hispanic White, 11 (26.2%) as Asian, six (14.3%) as Hispanic, three (7.1%) as Black, and one (2.4%) as multiracial. Moreover, 33 (78.6%) of the participants had a Bachelor’s or advanced degree, four (9.5%) attended some college, and five (11.9%) participants had a high school diploma or did not complete high school. The participants’ annual household income was grouped into five categories: Less than $25,000 (*n* = 6, 14.3%); between $25,000 and $35,000 (*n* = 4, 9.5%); between $35,000 and $50,000 (*n* = 4, 9.5%); between $50,000 and $75,000 (*n* = 8, 19.0%); and $75,000 or above (*n* = 19, 45.2%). One participant did not report their income. Participants’ religious affiliations included non-Catholic Christian (*n* = 30, 71.4%), Catholic Christian (*n* = 6, 14.3%), Jewish (*n* = 4, 9.5%), and other religions (*n* = 2, 4.8%). The sample characteristics are summarized in Table 1.

### 2.2. Interview Guide and Procedure

This study is part of a larger research project that assessed parental perceptions of ASD and genetic testing. To collect in-depth, comprehensive qualitative data from parents of children with ASD, semi-structured interview questions were developed based on past research [24,28,37] and modified for use in open-ended interviews. The validity of the interview guide was ensured by consulting experts in the fields of ASD, genetics, genomics, public health, special education, and qualitative research to evaluate the clarity and relevance of each interview question and to make further recommendations for changes. Pilot interviews were also conducted to evaluate the interview guide’s usability. We then refined the interview guide based on expert and pilot participant feedback. For example, we added probing questions, “Why” or “Why not,” to encourage participants to share more information. In the present study, we examined participating parents’ responses to two questions: (1) “What do you think caused your child to have ASD?” and (2) “Do you think there is any genetic factor causing it? Why or why not?

The interviews lasted approximately one hour and were conducted individually due to the sensitive nature of the topic and to protect participant privacy. The interview modalities included in-person/face-to-face (*n* = 21), telephone (*n* = 16), and Skype video conference (*n* = 5). Participants were informed about the purpose of the study, and their informed consent was obtained at the beginning of each interview. With participant approval, interviews were audio recorded with supplemental notetaking.

### 2.3. Data Analysis

After the interviews, we transcribed the recordings verbatim. Utilizing NVivo 12.0 (QSR International, Burlington, MA, USA), the transcribed data were coded and analyzed using the content analysis approach [38] following the method suggested by Zhang and Wildermuth [39]. Specifically, to develop a coding scheme, two coders (Z.Z. and H.W.) first coded 10 transcripts and categorized the codes into subthemes/themes using the constant comparative technique. The units of analysis were words, phrases, or sentences. Each code, subtheme, and theme in the scheme was clearly defined. Subsequently, we coded the rest of the interviews using the developed coding scheme. As this process continued, newly emerged codes and themes from the data were added to the coding scheme.

To establish the trustworthiness of our study, both method and investigator triangulation were applied [40]. In addition to the transcripts, the field notes taken during the interviews were used to facilitate coding and interpretation. Moreover, the coding was consistent between the two coders (>90%), and any discrepancies were reviewed, discussed, and recoded jointly to reach consensus. A third researcher with expertise in ASD, genomics, and qualitative research (L.-S.C.) reviewed the codes, sub-themes, and themes to confirm the accuracy of the coding.

## 3. Results

We conducted in-depth, semi-structured interviews in which participating parents were asked what they perceived to be the cause of their children’s ASD. Among the 42 participants, 17 (40.5%) mentioned genetic factors, 13 (31.0%) mentioned environmental factors, 10 (23.8%) thought it might be related to problems that occurred during the mother’s pregnancy and delivery, seven (16.7%) thought the cause might be vaccination, three (7.1%) mentioned their child’s non-ASD-related health problems, two (4.8%) thought that their age at the time of pregnancy might be the cause, and one (2.4%) participant believed that spiritual or religious factors might be involved. Four (9.5%) participants were unsure about or did not know the causes of their child’s ASD. The results are summarized in Table 2.

The following sections describe the seven themes identified via content analysis in regards to the parental perceptions of ASD etiology and underlying reasons. The sections are ordered from the most frequently discussed perceived causes of ASD to the least cited reason for ASD: Genetic factors, environmental factors, problems that occurred during pregnancy or deliver, vaccination, other health problems, parental age at the time of pregnancy, and spiritual or religious factors.

### 3.1. Genetic Factors

There were 17 participants (40.5%) who believed that genes, DNA (deoxyribonucleic acid), or a vulnerability in the “genetic system” might be the root cause of their children’s ASD. One participant said that he had his son genetically tested, and the test found a genetic anomaly that was highly correlated to ASD. As described by this father who held a doctorate:

“I think he has a genetic anomaly that affects the biochemical processes in some number of his cells and therefore, probably affects the neurological connections in the brain at some point.”

This participant also mentioned that his family health history showed that several members of his family had autistic tendencies:

“You can see autistic tendencies in myself: I’m very good at blocking out the rest of the world while I’m doing an activity when I’m into it… My wife’s brother can be socially awkward; he misses social cues, which is somewhat related to autism.”

Similarly, another participant reported that both she and her husband had multiple family members who exhibited forms of ASD or mental retardation. According to her:

“I think it’s a combination of my husband and I. On my side I have an uncle who is schizophrenic and a sister who has ADD [Attention Deficit Disorder], my husband is bipolar, he has another uncle who they thought might be bipolar as well but maybe he was autistic.”

One participant also thought that genetic factors were the primary reason because she noticed similarities between her husband’s and her son’s speech. Her husband liked to use repetitive phrases; specifically, he would repeat the same statements over and over again to bridge social communication when he did not know what to say. Similar to his father, the son, who was diagnosed with ASD, would sometimes repeat the same high-pitched noise over and over again to self-stimulate. Thus, the participant believed that her son’s ASD was related to her husband’s genes.

### 3.2. Environmental Factors

Almost one-third (31.0%) of the participants believed that environmental factors led to their children’s ASD. They were concerned about the chemicals in food, water and air pollution, as well as poisonous chemicals in the environment. One participant told us that she took her son to a pediatric neurologist when he was in the fifth grade, and the pediatric neurologist informed her that her son’s condition looked more like heavy metal poisoning. According to her:

“We did live in a neighborhood that was polluted by arsenic. We moved in about a month before he was born, so his early childhood was in that neighborhood with arsenic pollution.”

In addition, one participant explained that she was in graduate school during her pregnancy, and her research project involved exposure to phenol, which is teratogenic. Due to following incorrect procedure, this participant found that she had not opened the fume hood until half an hour after she began working. She believed that this might have contributed to her child’s ASD.

### 3.3. Problems during the Mother’s Pregnancy and Delivery

There were 10 participants (23.8%) who described the problems they experienced during pregnancy and delivery as the causes of their children’s ASD. Several participants believed that the medications they took during pregnancy, such as fertility drugs and drugs for treating flu, might have led to the development of their children’s ASD. A mother reported that she almost miscarried due to hemorrhage and cramps during pregnancy.

A mother mentioned that as she had a contraceptive device in her body, she did not know that she was pregnant until two months into the pregnancy, during which this participant’s physical and mental states were unstable. Thus, she believed that the instability might have affected the development of the fetus and caused her child’s ASD.

Some parents believed that their children’s ASD or developmental abnormalities were caused by a lack of oxygen in the brain when they were born prematurely. As described by a mother:

“She was born at 26 weeks, so extremely premature. And we had been told very early on that she was at a high risk for not only developmental delays, but also mental retardation, autism and cerebral palsy, all of those things.”

Other causal factors identified by parents included morning sickness, insufficient nutrition during pregnancy, long labor time, and anesthesia or forceps being used during childbirth.

### 3.4. Vaccination

Seven (16.7%) participants attributed the cause of their child’s ASD to vaccinations. Some parents believed that a RhoGAM shot, as given to the mother during pregnancy, might have affected the fetus. They also mentioned that the MMR vaccine could be the reason for their children’s ASD. According to them, their children showed signs of a slower response after receiving the MMR vaccine. A mother attributed the etiology of her daughter’s ASD to the MMR vaccine because no one in her or her husband’s family has ASD or similar symptoms. Another participant clearly stated that the vaccine led to her child’s ASD. According to her:

“So, at six months he had a shot and nothing happened. But at nine months he had another shot and then 12 months it was a big shot that, oh, my, God, that was…wow. He had a look, really sleepy and like tired all day long.”

In addition, one participant thought that her son probably had a predisposition to ASD, and the vaccines he received made his condition worse.

### 3.5. Non-ASD-Related Health Problems

Three (7.1%) of the participants cited their children’s non-ASD-related health problems as the likely cause of ASD, including a kind of brain stroke, digestive problems, and sensitive immune systems. For example, one mother told us that her son’s digestive problems have a lot to do with his overall development. According to this mother:

“Yeah, well, all of his digestive problems, I think, have a lot to do with his development in general. He was on a lot of antibiotics when he was a baby.”

### 3.6. Parental Age at the Time of Pregnancy

Two participants (4.8%) perceived that age at the time of pregnancy was the contributing ASD factor. A mother, who worked in academia, stated that they conceived their child through in vitro fertilization (IVF), and that both she and her husband were a little bit old at the time of her pregnancy. The other participant, a father with a doctorate, mentioned that he was old when his wife became pregnant. Thus, he believed that parental age could be a cause of ASD.

### 3.7. Spiritual or Religious Factors

One participant (2.4%) perceived that spiritual or religious factors were the potential cause of ASD. This participant claimed that supernatural factors, such as destiny, karma, or a curse, might be associated with the development of ASD. The participant further explained that if someone had carried out a bad deed, such as murder, this would lead to a curse (or bad karma) causing harm to this individual’s family, such as having a child with ASD.

## 4. Discussion

To better serve families affected by ASD, it is essential to understand what parents perceive as the cause of ASD in their children. The present study examined the perceived causes of ASD among parents of children diagnosed with ASD via qualitative data obtained from in-depth, semi-structured interviews. Several ASD etiologies, as perceived by participating parents, were identified in this study. Consistent with past research [24,28,36], the perceived causes of ASD included genetic factors, environmental factors (e.g., water or air pollution), problems that occurred during pregnancy or delivery (e.g., lack of oxygen in the child’s brain), vaccinations (e.g., MMR vaccine), other health problems (e.g., compromised immune system), parental age at the time of pregnancy, and spiritual or religious factors.

Surprisingly, the percentage of our participating parents who perceived genetics as the cause of ASD (40.5%) was lower than those of past research: 90.2% in Mercer et al.’s work [24] and 72.6% in Selkirk et al.’s study [28]. This discrepancy may be related to demographic differences among the studies as the percentage of non-Hispanic White/Caucasian participants (50.0%) in the present study was smaller than in Mercer et al.’s (90.2%) [24] or Selkirk et al.’s (93.7%) works [28]. Research has suggested that ethnic or racial disparities in ASD-related health services, education, and campaigns [22,25] may be associated with parental perceptions of ASD etiology. More research may be needed to examine the differences in ASD perceptions among different ethnic or racial groups. In alignment with past literature, however, genetic factors or influences were cited as the most commonly perceived cause of ASD, followed by environmental factors (e.g., environmental pollution or toxins) [24,28].

As expected, despite the fact that scientific research has debunked vaccine-related misconceptions and reported no evidence supporting any link between vaccines and ASD [17,21], some parents still perceived vaccines as a cause of ASD. This finding is consistent with past research [24,28,30]. Mercer et al. found that among their participants who attributed their child’s ASD to vaccines, 32.5% believed that the MMR vaccine was responsible [24]. These perceptions or misconceptions may be associated with confirmatory bias during internet searches and misinformation available online that suggests a link between vaccines and ASD development without supporting scientific evidence [16,41]. Efforts should be continued to diminish and eventually eliminate the misconception that vaccination is a cause of ASD.

Some limitations of the present study should be noted. First, while we recruited parents of children with ASD from various professional health conferences and ASD communities, participants self-reported their children’s ASD diagnoses. We did not verify these diagnoses using medical records. Second, due to the qualitative nature of the study, our sample size may have been small compared to some studies. However, we reached data saturation at 42 participant interviews, which is sufficient for qualitative research [38]. Lastly, although we aimed to recruit a diverse sample, most of our participating parents reported high socioeconomic status, which may limit the generalizability of the findings. Yet, this demographic is consistent with past research involving parents of children diagnosed with ASD [2,28,30]. Future research should consider targeting parents with lower socioeconomic status.

## 5. Conclusions

A crucial step in providing support, education, and guidance to families affected by ASD is to understand how parents perceive the cause of ASD. Parental perceptions can affect parent–child bonding and relationships with healthcare providers. Parental perceptions of ASD etiology can also affect their child’s ASD prognosis, treatment, and management, as well as reproductive and family planning decisions [2,24,25]. We identified a diverse range of parental views pertaining to ASD etiology in our sample. Some factors, however, had no scientific basis. In particular, misconceptions about vaccinations as the cause of their child’s ASD can negatively influence parental health behaviors (e.g., vaccinating their children and younger siblings) and prevent health professionals from providing optimal ASD-related services, education, and treatment [2,16,23]. Researchers and health professionals can use the findings of the present study to develop and provide targeted education to families of children with ASD. The results of our study also have the potential to support policymakers in developing campaigns that increase parental ASD awareness and knowledge.

## Figures and Tables

**Table 1:** Sample characteristics.

Variable	*M* (*SD*)/*n* (%)
Age	*M* = 44.3, *SD* = 8.5, range = 24–58
Parent	
Mothers	32 (76.2%)
Fathers	10 (23.8%)
Ethnicity	
Non-Hispanic White	21 (50.0%)
Asian	11 (26.2%)
Hispanic	6 (14.3%)
Black	3 (7.1%)
Multiracial	1 (2.4%)
Education Level	
Bachelor’s or advanced degree	33 (78.6%)
Some college	4 (9.5%)
High school diploma or below	5 (11.9%)
Annual Household Income ^1^	
Less than $25,000	6 (14.3%)
Between $25,000 and $35,000	4 (9.5%)
Between $35,000 and $50,000	4 (9.5%)
Between $50,000 and $75,000	8 (19.0%)
$75,000 or above	19 (45.2%)
Religion	
Non-Catholic Christian	30 (71.4%)
Catholic Christian	6 (14.3%)
Jewish	4 (9.5%)
Other	2 (4.8%)
Number of children diagnosed with ASD	
One child	32 (76.2%)
Two children	9 (21.4%)
Three children	1 (2.4%)

*M*, mean; *SD*, standard deviation; *n*, group size; %, percentage; *ASD*, Autism spectrum disorder. ^1^ One participant did not report their income.

**Table 2:** Parents’ Perceived Causes of ASDs.

Parents’ Perceived Causes	Frequency	Percentage	Illustrative Quote
Genetic factors	17	40.5%	“I think that there is a genetic predisposition that is happening to families. And so yes I think that there is something genetic that’s at least in our family.”
Environmental factors	13	31.0%	“I think there are pollutants that are a part of it and maybe proximity to certain types of industry can add to it.”
Problems during the mother’s pregnancy and delivery	10	23.8%	“Her [the child with ASD’s] tongue is a little bit too malformed and her jaw is malformed. So I’m thinking something happened to her when I was pregnant.”
Vaccination	7	16.7%	“I really think it’s vaccine related because in my family and my husband’s family you know family history, we don’t we have anybody you know in that category.”
Children’s non-ASD-related health problems	3	7.1%	“We wonder if there was possibly some kind of a brain stroke.”
Parental age at the time of pregnancy	2	4.8%	“The father’s age was older. The people I can point out right now were all over 40 then.”
Spiritual or religious factors	1	2.4%	“I am not sure if you have. It can’t be studied scientifically. If we are talking about reasons, in terms of religious beliefs, I think it’s a curse from ancestors.”

Note: Four participants (9.5%) were unsure about or did not know the causes of ASD. The total percentage was more than 100%, as some parents identified more than one contributing factor for their children with ASD.

## Data Availability

The data are not publicly available as data sharing was not included within the original study ethics submission or participant consent form.
